# Retraction: A Rare Case of Disseminated Varicella Zoster Virus (VZV) Infection Complicated by VZV Pneumonia

**DOI:** 10.7759/cureus.r185

**Published:** 2025-06-03

**Authors:** Suhel A Sabunwala, Tuhina Cornelius

**Affiliations:** 1 Infectious Diseases, Methodist South Hospital, Memphis, USA

This article has been retracted by the Editors-in-Chief due to the inclusion of identifiable patient images without documented written informed consent. The images of the patient have been removed from the retracted article. The journal greatly regrets that written informed consent was not obtained and verified prior to publication.

